# Pain management after abdominal surgery: requiem for epidural analgesia?

**DOI:** 10.1093/bjs/znae299

**Published:** 2024-11-27

**Authors:** Dileep N Lobo, Girish P Joshi

**Affiliations:** Nottingham Digestive Diseases Centre, Division of Translational Medical Sciences, School of Medicine, University of Nottingham, Queen's Medical Centre, Nottingham NG7 2UH, UK; National Institute for Health Research (NIHR) Nottingham Biomedical Research Centre, Nottingham University Hospitals NHS Trust and University of Nottingham, Queen’s Medical Centre, Nottingham, UK; MRC Versus Arthritis Centre for Musculoskeletal Ageing Research, School of Life Sciences, University of Nottingham, Queen’s Medical Centre, Nottingham, UK; Division of Surgery, Perelman School of Medicine, University of Pennsylvania, Philadelphia, Pennsylvania, USA; Department of Anesthesiology and Pain Management, University of Texas Southwestern Medical Center, Dallas, Texas, USA

Three factors that delay discharge from hospital for patients undergoing major abdominal surgery are poorly managed pain, inability to eat and drink, and impaired mobility. Continuous epidural analgesia has been a major component of enhanced recovery surgery programmes for abdominal surgery since the first guidelines were published in 2005^1^, as it results in better pain control which may improve breathing and mobility. In addition, the resulting sympathetic blockade reduces the duration of postoperative ileus, and may enable the patient to eat and drink earlier. However, continuous epidural analgesia has drawbacks. In the real world, it works well in only 60–70% of patients^2^, and it can cause urinary retention and hypotension, the latter frequently being treated with fluid infusion, resulting in salt and water overload and the associated adverse events^3^.

In this issue of *BJS*, Lee *et al.*^4^ have presented the results of a non-inferiority RCT comparing continuous preperitoneal infusion of ropivacaine, a long-acting local anaesthetic, with thoracic epidural analgesia in patients undergoing open pancreatoduodenectomy via a midline incision. Epidural analgesia was under patient control and the analgesics used were ropivacaine and fentanyl. Both epidural and wound catheters were left *in situ* until postoperative day 3. The investigators found no difference in mean pain scores both at rest and on coughing on postoperative days 1 and 2. However, the continuous wound infusion group had significantly better pain relief and quality of recovery on postoperative day 3. Postoperative hypotension was more frequent in the thoracic epidural group, but total opioid consumption and time to the first passage of flatus were significantly lower in this group. There were no differences between the groups when other endpoints, including nausea and vomiting, postoperative complications and duration of hospital stay, were considered. However, the authors did not report on ambulation and tolerance of oral feeding.

One of the strengths of this study was that epidural catheters were inserted under fluoroscopic guidance; this has been shown to result in correct placement of the catheter in the epidural space in up to 98% of instances, compared with 74% when the operator is dependent on loss of resistance with air or saline^5^, because fluoroscopy confirms the epidural position, assesses catheter level and spread pattern, and can identify anatomical variations, and coiling or kinking of the catheter^2^. This reduced the risk of malfunctioning epidurals and, perhaps, had the catheter placement been dependent on loss of resistance, the investigators may have shown that epidural analgesia was inferior to continuous wound infusion. Preperitoneal space catheters were also placed under direct vision, thereby ensuring that they were in the correct plane.

Another strength of this study is that it followed most of the essential features of a non-inferiority study design^6^, including the use of a per-protocol approach as well as intention-to-treat analysis to ascertain reliability. In addition, the study seems to have selected an appropriate non-inferiority margin based on previously published data and clinically meaningful differences in pain scores. The investigators also followed procedure-specific postoperative pain management methodology by predetermining that a difference in visual analogue scale pain scores of at least 1 was necessary to demonstrate clinically relevant differences between the groups^7^.

One of the limitations of this study is that, although paracetamol was administered every 8 h, ketorolac was given as rescue medication. However, it is recommended that non-steroidal anti-inflammatory drugs (NSAIDs) or cyclo-oxygenase (COX) 2-specific inhibitors are administered round the clock (that is as scheduled)^7^. The combination of paracetamol and NSAIDs or COX-2 specific inhibitors has been shown to be more effective than either drug alone^7^. It is possible that the scheduled administration of NSAIDs or COX-2-specific inhibitors would have improved pain relief and reduced opioid consumption. Furthermore, the greater opioid use in the preperitoneal infusion group may have been because the epidural group received a combination of ropivacaine and fentanyl, whereas the continuous wound infusion group received ropivacaine alone. Epidurally administered fentanyl has been shown to be equivalent to intravenous administration of fentanyl, and may be a reason for reduced total opioid consumption in the epidural group.

Another limitation of this study is that the management of both study groups did not conform to optimal clinical practice. Given that dynamic pain was moderate to severe (visual analogue scale pain scores of 5.7–6.8), the authors should have allowed for modifications in the infusion rate and/or patient-controlled settings (patient bolus and lockout period) in the epidural group, and allowed for an increase in the basal infusion rate in the preperitoneal group. Notably, a ropivacaine infusion rate of 2 ml/h in the preperitoneal space is usually inadequate to achieve sufficient pain relief.

The use of continuous wound infusion for major abdominal surgery is not new; a recent meta-analysis^8^ of 3 RCTs and 5 observational studies, with 484 participants, showed that, although thoracic epidural analgesia reduced the requirement for additional intravenous analgesia compared with rectus sheath catheters after laparotomy, both techniques were equivalent when pain scores were compared. Furthermore, participants with rectus sheath catheters had a lower rate of postoperative hypotension and ambulated earlier^8^. Another meta-analysis^9^ comparing thoracic epidural analgesia with transversus abdominis plane blocks after colorectal surgery showed that, although postoperative pain control was similar in both groups, transversus abdominis plane blocks allowed better functional recovery with a lower incidence of adverse events.

A meta-analysis^10^ that included 3 RCTs and 8 cohort studies, with 25 089 participants, compared epidural analgesia with non-epidural techniques (intravenous morphine, continuous wound infiltration, bilateral paravertebral thoracic catheters, and intrathecal morphine) after pancreatoduodenectomy. Participants receiving epidural analgesia had similar pain scores to those who had continuous wound infiltration. Treatment failure, mainly due to haemodynamic instability and inadequate pain control, occurred in 28.5% of patients receiving epidural analgesia^10^.

Although peripheral regional anaesthesia techniques, such as preperitoneal local anaesthetic infusion and interfascial plane blocks, are increasingly replacing epidural anaesthesia, the latter still has a role in patients who are at high risk of postoperative pain (for example, high pain responders), and those in whom basic analgesics cannot be administered. In addition, meticulous and systematic surgical-site local anaesthetic infiltration in the peritoneal, musculofascial, and subdermal tissues before wound closure may further add to the analgesic benefits of preperitoneal local anaesthetic infusion.

In summary, despite some of the limitations, the study by Lee *et al.*^4^ has provided further evidence that continuous wound infusion in the correct plane with a local anaesthetic is equivalent to epidural analgesia. Increased adoption of these techniques could obviate the issue of non-functioning epidurals and the potential complications of epidurals, including hypotensive episodes.
